# Assembly and comparative analysis of the complete mitochondrial genome of the *Maclura tricuspidata*

**DOI:** 10.1186/s12864-025-12491-z

**Published:** 2026-01-05

**Authors:** Shengkui Zhang, Xia Wang, Xianyan Zhao, Ziyang Gao, Kun Pan

**Affiliations:** 1 School of Bioengineering, Qilu University of Technology (Shandong Academy of Sciences), Jinan, Shandong China; 2 School of Architecture and Design, Chongqing College of Humanities, Science and Technology, Chongqing, China; 3 School of Pharmacy, Hainan Medical University, Haikou, Hainan China

**Keywords:** Maclura tricuspidata, Mitochondrial genome, Genome annotation, Comparative genomics, RNA editing

## Abstract

**Background:**

*Maclura tricuspidata* is an important medicinal and horticultural plant. However, the complete mitochondrial genome (mitogenome) of *M. tricuspidata* has not been reported, hindering molecular phylogenetic studies, species identification, and evolutionary research.

**Results:**

We present the first comprehensive analysis of the *M. tricuspidata* mitogenome. It features a multi-chromosomal structure comprising three circular-mapping chromosomes, with a total length of 416,801 bp and a GC content of 44.94%. Annotation identified 28 unique protein-coding genes (PCGs), 18 tRNA genes, and 3 rRNA genes. Codon usage analysis revealed GCU and CAA as the predominant codons for alanine and glutamine, respectively, while methionine and tryptophan, as single-codon amino acids, showed no bias. A total of 154 simple sequence repeats (SSRs) were detected: 84 on chromosome 1, 48 on chromosome 2, and 22 on chromosome 3. Analysis identified 19 homologous fragments transferred from the chloroplast genome (cpDNA), accounting for 4.31% of the mitogenome length. Using the Deepred-mt suite, 409 C-to-U RNA editing sites were predicted from the complete set of 28 mitochondrial PCGs, with the highest number in *nad4* and the lowest in *sdh4*. Phylogenetic analysis confirmed the placement of *M. tricuspidata* within the Moraceae family, showing closest relationships to *Ficus carica* and *Morus notabilis*, consistent with the APG IV system. Comparative analysis revealed extensive syntenic blocks between the *M. tricuspidata* mitogenome and those of related species, alongside regions lacking homology. In addition, dN/dS analysis revealed that most of the protein-coding genes in the mitogenome had undergone negative selection, and only the *ccmB* and *sdh4* gene had undergone potential positive selection in *M. tricuspidata*.

**Conclusions:**

The unique structural features and complexities of the *M. tricuspidata* mitogenome, along with its similarities and differences compared to related species, provide valuable insights into plant mitochondrial evolution, energy metabolism, and environmental adaptation. These findings contribute significantly to the understanding of plant mitogenome diversity and biology.

**Supplementary Information:**

The online version contains supplementary material available at 10.1186/s12864-025-12491-z.

## Background

Plant mitochondrial genomes (mitogenomes) represent dynamic and structurally complex components of eukaryotic cells, playing indispensable roles beyond their primary function in oxidative phosphorylation and ATP synthesis [1, 2]. Recent advances, propelled by long-read sequencing and advanced imaging techniques, have fundamentally challenged the historical paradigm of mitogenomes as simple circular monomers. Instead, they are now recognized as polymorphic entities, frequently adopting linear, branched, or multicircular subgenomic conformations [3, 4]. For instance, multichromosomal structures, consisting of several independent circular DNA molecules, have been confirmed in various plant species such as cucumber (*Cucumis sativus*) and poplar (*Populus deltoides*) [5, 6]. This remarkable structural plasticity arises from extensive genomic rearrangements, pervasive intramolecular recombination mediated by repetitive sequences, frequent gene duplication or loss, and ongoing intracellular gene transfer (IGT) events [7, 8]. Consequently, mitogenome size exhibits extraordinary variation across land plants, ranging from the highly compacted 66-kb genome in *Viscum scurruloideum* to expansive genomes exceeding 4 Mb in certain gymnosperms [9, 10]. While the ancestral angiosperm mitogenome likely encoded 41 protein-coding genes (PCGs), extant species display significant variation in gene content due to pseudogenization, functional transfer to the nucleus, and lineage-specific modifications [11, 12].

The intricate architecture of plant mitogenomes is intrinsically linked to their critical biological functions. Beyond energy production, mitogenomes are essential regulators of plant development, growth, differentiation, and programmed cell death [1, 13]. Their predominantly maternal inheritance pattern simplifies genetic analyses by eliminating paternal contributions [14]. Crucially, the inherent structural dynamism of mitogenomes, particularly recombination-driven formation of novel chimeric open reading frames (ORFs), underpins cytoplasmic male sterility (CMS) – a phenomenon widely harnessed in hybrid crop breeding [15, 16]. However, this very complexity, characterized by repetitive elements, subgenomic circles, and multi-chromosomal organizations (e.g., cucumber) [5, 17], presents formidable challenges for achieving complete and accurate de novo assembly using traditional short-read technologies [18]. Studies relying solely on such methods have often oversimplified mitogenomes as circular maps [19, 20], a misconception that risks hindering molecular breeding efforts and obscuring the true mechanisms of traits like CMS [16, 21]. Therefore, elucidating the genuine physical structure, including potential bifurcating configurations, using modern long-read sequencing approaches is imperative for advancing our understanding of plant mitochondrial genetics, evolution, and its applications in agriculture [3, 6, 8].

*Maclura tricuspidata* (syn. *Cudrania tricuspidata*) is a significant species within the Moraceae family [22]. Primarily distributed across northern, eastern, southwestern, and central-southern China, it thrives on sunny hillsides, forest margins, and swampy areas at elevations of 500–1500 m [23]. This species holds considerable economic and ecological value. Its stem bark fibers are traditionally used in papermaking, the root bark possesses medicinal properties, and the fruits are edible or used in winemaking. The dense, yellow heartwood is suitable for furniture and yields a natural dye [22, 24].

*M. tricuspidata* has a long history in traditional medicine. In China, its roots (“Chuan-Po-Shi”) and root bark are used to treat conditions such as gonorrhea, jaundice, rheumatism, contusions, hemoptysis, and lumbago [25, 26]. Notably, it has gained prominence in South Korea as a key ethnomedicinal plant for cancer therapy [27]. Extracts demonstrate significant pharmacological activities against inflammation, tumors, obesity, and diabetes, with flavonoids and xanthones identified as major bioactive constituents [27, 28]. Despite its economic and medicinal importance, molecular biology and genomic studies on *M. tricuspidata* remain limited. Key aspects such as its regulatory networks, functional genes, and genomic architecture, particularly the mitogenome, are yet to be comprehensively characterized.

Here, we report the first complete sequence and assembly of the *M. tricuspidata* mitogenome, featuring comprehensive annotation, codon usage and repeat sequence analyses, comparative genomics, assessment of chloroplast-derived DNA transfer, RNA editing prediction, and phylogenetic and synteny investigations. This foundational resource expands the available genetic information on this important species and provides crucial insights for its future genetic improvement.

## Results

### Structure features and annotation of the *M. tricuspidata* mitogenome

**Fig. 1:**
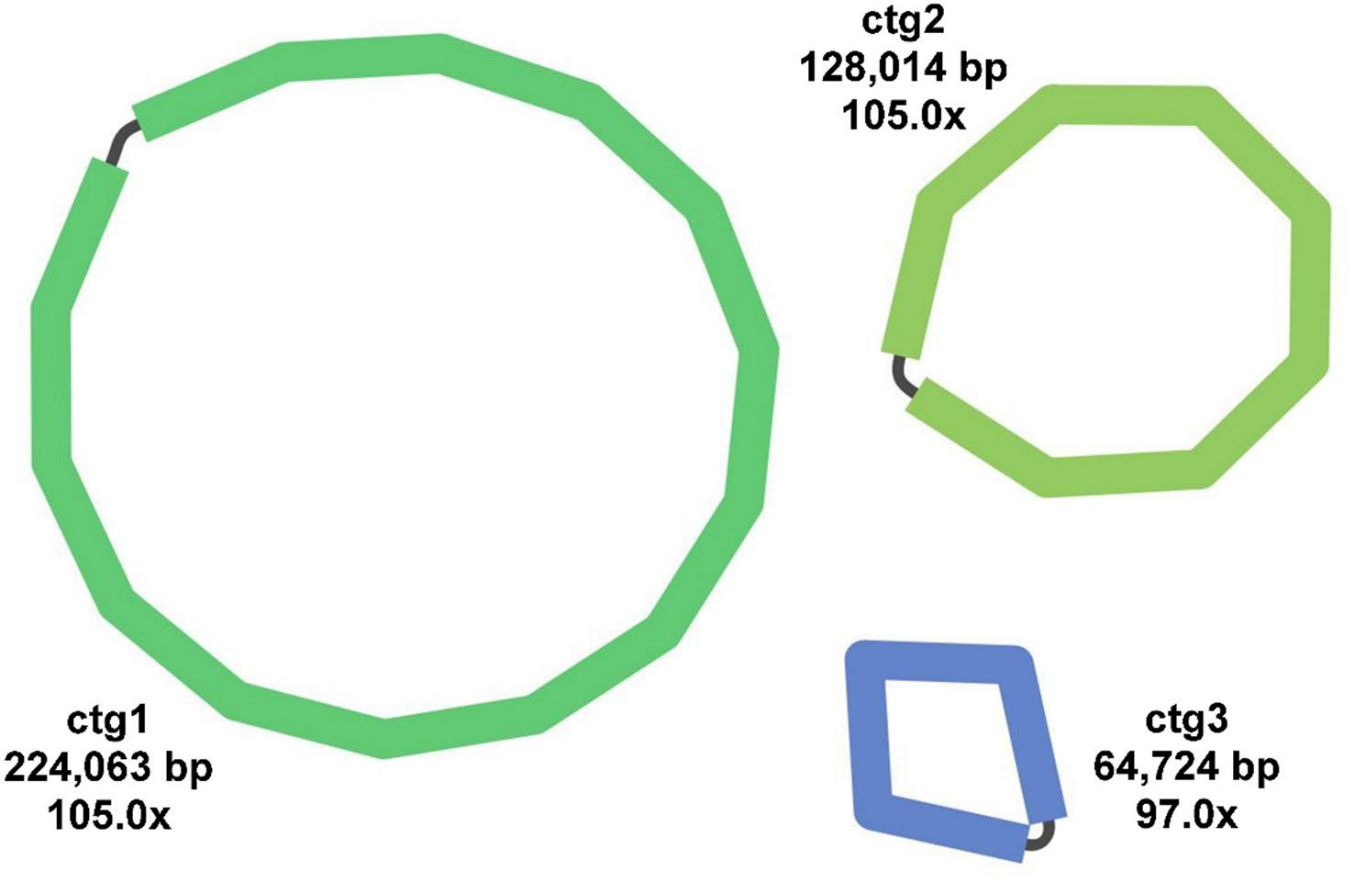
Structural organization of the *M. tricuspidata* mitogenome showing three circular-mapping molecules

The mitochondrial genome of *M. tricuspidata* was assembled into three circular chromosomes (Chromosome 1–3) with a combined length of 416,801 bp and an overall GC content of 44.94%. This multi-chromosomal structure was consistently resolved by independent assemblies using both Flye and HiFiasm, based on PacBio HiFi sequencing data (~ 203× coverage) with duplicated regions removed, thereby providing robust cross-validation. The assembly exhibits high continuity, as evidenced by an N50 of 224,063 bp and an L50 of 1, with the three contigs measuring 224,063 bp (GC 45.37%), 128,014 bp (GC 44.49%), and 64,724 bp (GC 44.34%), respectively (Fig. 1). The consistency between assemblers, coupled with these metrics, strongly supports the accuracy and reliability of the mitogenome assembly.

**Fig. 2:**
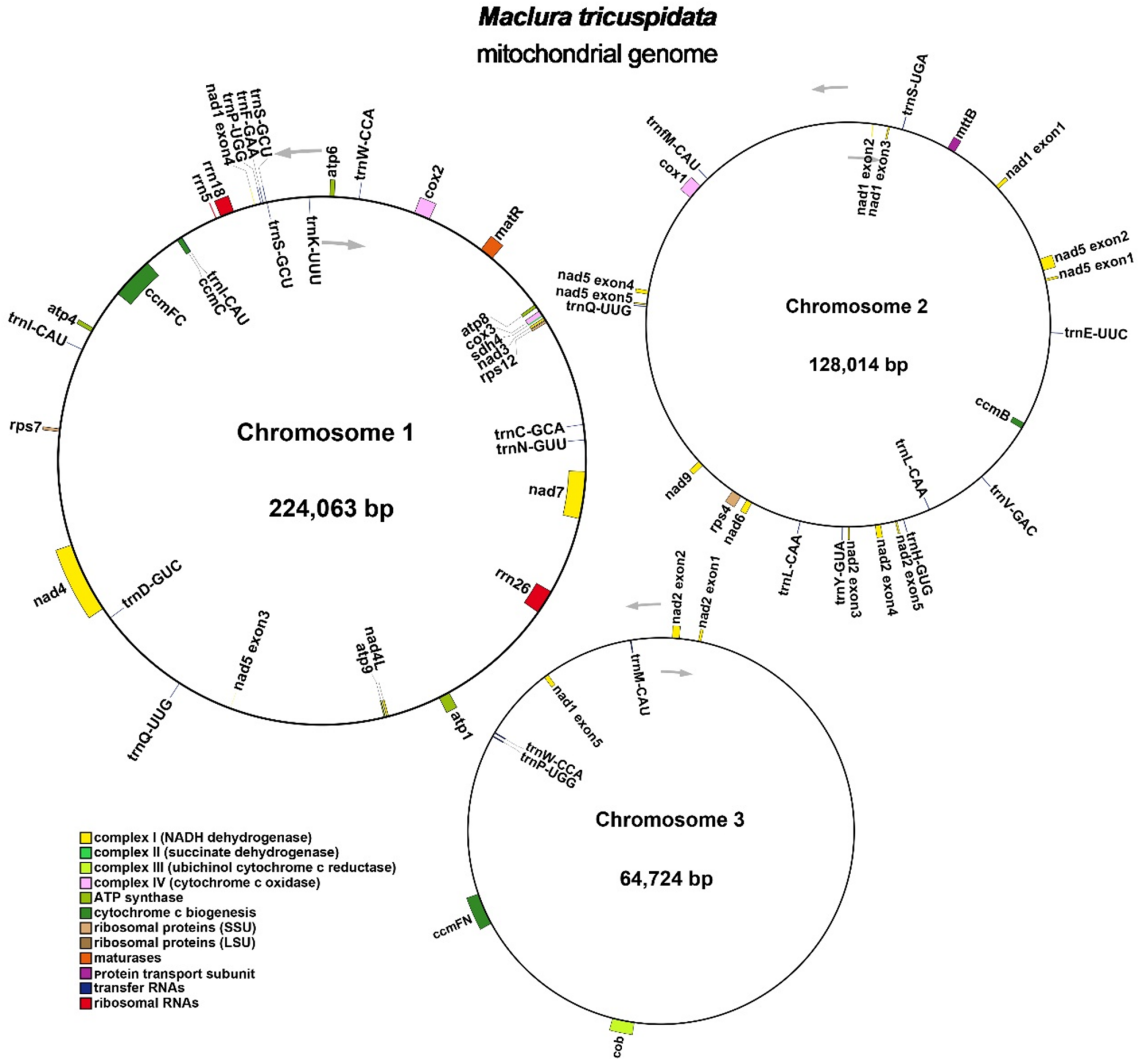
*M. tricuspidata* mitogenome gene map. Genes located inside the circle are transcribed in a clockwise direction, while those outside the circle are transcribed counterclockwise

Annotation of the mitogenome identified 28 distinct protein-coding genes (PCGs), including 24 conserved mitochondrial core genes and 4 accessory genes, along with 18 transfer RNA (tRNA) genes (among which 6 are multi-copy) and 3 ribosomal RNA (rRNA) genes (Table 1; Fig. 2). The core PCGs comprised: five ATP synthase subunits (*atp1*, *atp4*, *atp6*, *atp8*, *atp9*); nine NADH dehydrogenase subunits (*nad1*, *nad2*, *nad3*, *nad4*, *nad4L*, *nad5*, *nad6*, *nad7*, *nad9*); four cytochrome c biogenesis proteins (*ccmB*, *ccmC*, *ccmFC*, *ccmFN*); two cytochrome c oxidase subunits (*cox1*, *cox3*); one membrane transport protein (*mttB*); one maturase (*matR*); one apocytochrome b (*cob*). The accessory PCGs consisted of three ribosomal small subunit proteins (*rps4*, *rps7*, *rps12*) and one succinate dehydrogenase subunit (*sdh4*).

**Table 1:** Gene annotation of the *M. tricuspidata* mitogenome

Group of genes	Name of genes
ATP synthase	*atp1*,* atp4*,* atp6*,* atp8*,* atp9*
NADH dehydrogenase	*nad1*,* nad2*,* nad3*,* nad4*,* nad4L*,* nad5*,* nad6*,* nad7*,* nad9*
Cytochrome b	*cob*
Cytochrome c biogenesis	*ccmB*,* ccmC*,* ccmFC*,* ccmFN*
Cytochrome c oxidase	*cox1*,* cox2*,* cox3*
Maturases	*matR*
Protein transport subunit	*matB*
Ribosomal protein small subunit	*rps4*,* rps7*,* rps12*
Succinate dehydrogenase	*sdh4*
Ribosome RNA	*rrn5*,* rrn18*,* rrn26*
Transfer RNA	*trnC-GCA*,* trnD-GUC*,* trnE-UUC*,* trnF-GAA*,* trnM-CAU*,* trnH-GUG*,* trnI-CAU (×2)*,* trnK-UUU*,* trnL-CAA (×2)*,* trnM-CAU*,* trnN-GUU*,* trnP-UGG (×2)*,* trnQ-UUG (×2)*,* trnS-GCU (×2)*,* trnS-UGA*,* trnV-GAC*,* trnW-CCA (×2)*,* trnY-GUA*

The numbers in brackets represent the copy number of genes. For example, (×2) means there are two copies

### Codon usage analysis of PCGs in *M. tricuspidate*

**Fig. 3:**
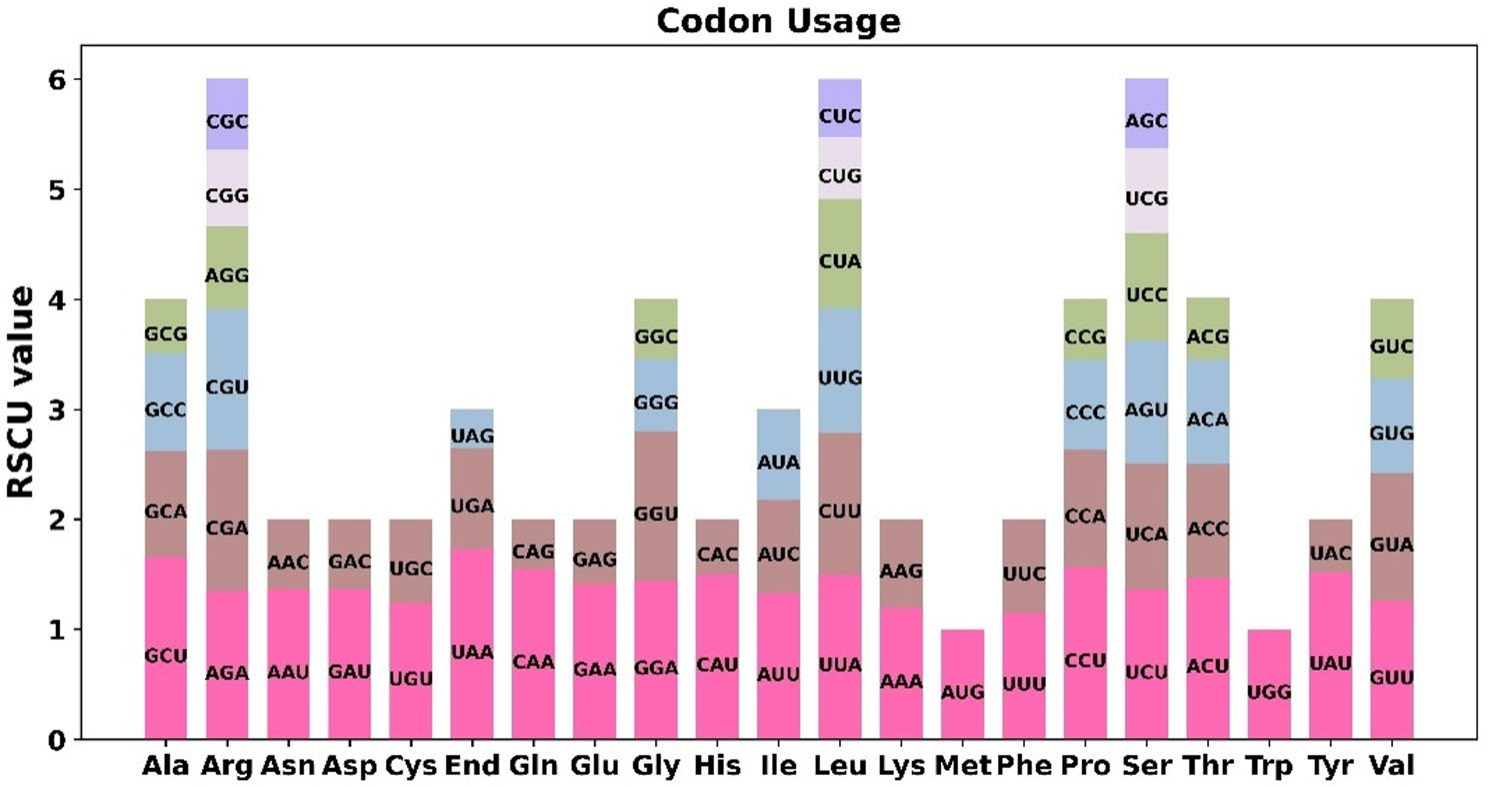
Analysis of codon usage bias in *M. tricuspidata* mitogenomes. X-axis, codon families; Y-axis, the relative synonymous codon usage (RSCU) value. RSCU measures the likelihood of a specific codon being used among synonymous codons that encode the same amino acid and values greater than 1 indicate a higher frequency of usage for the codon

The codon usage of 28 unique PCGs from *M. tricuspidate* was analyzed, and those with a relative synonymous codon usage (RSCU) greater than 1 were considered to be preferentially used by amino acids. Beyond the start codon AUG (Met) and UGG (Trp) - both exhibiting RSCU values of 1 - widespread codon usage bias was also observed among mitochondrial PCGs. (Fig. 3; Table S1). Stop codon UAA showed extreme preference (RSCU = 1.73), while UAG was strongly avoided (0.35). Arginine exhibited triple-codon bias with AGA (1.35), CGA (1.28) and CGU (1.28) preferred over CGG (0.70). Alanine demonstrated GCU dominance (1.66) versus GCG avoidance (0.49). A/U-ending codons dominated: AAU (Asn, 1.36), GAU (Asp, 1.37), CAA (Gln, 1.55), UUA (Leu, 1.50), and UAU (Tyr, 1.52). Contrastingly, G/C-ending counterparts showed suppression (e.g., AAC = 0.64, CAG = 0.45). Proline displayed CCU preference (1.57) but CCG avoidance (0.55). Methionine (AUG) and tryptophan (UGG) showed no bias as single-codon amino acids. These patterns reflect strong A/U nucleotide preference at synonymous codon third positions.

### Repeat sequence analysis of mitogenomes

**Fig. 4:**
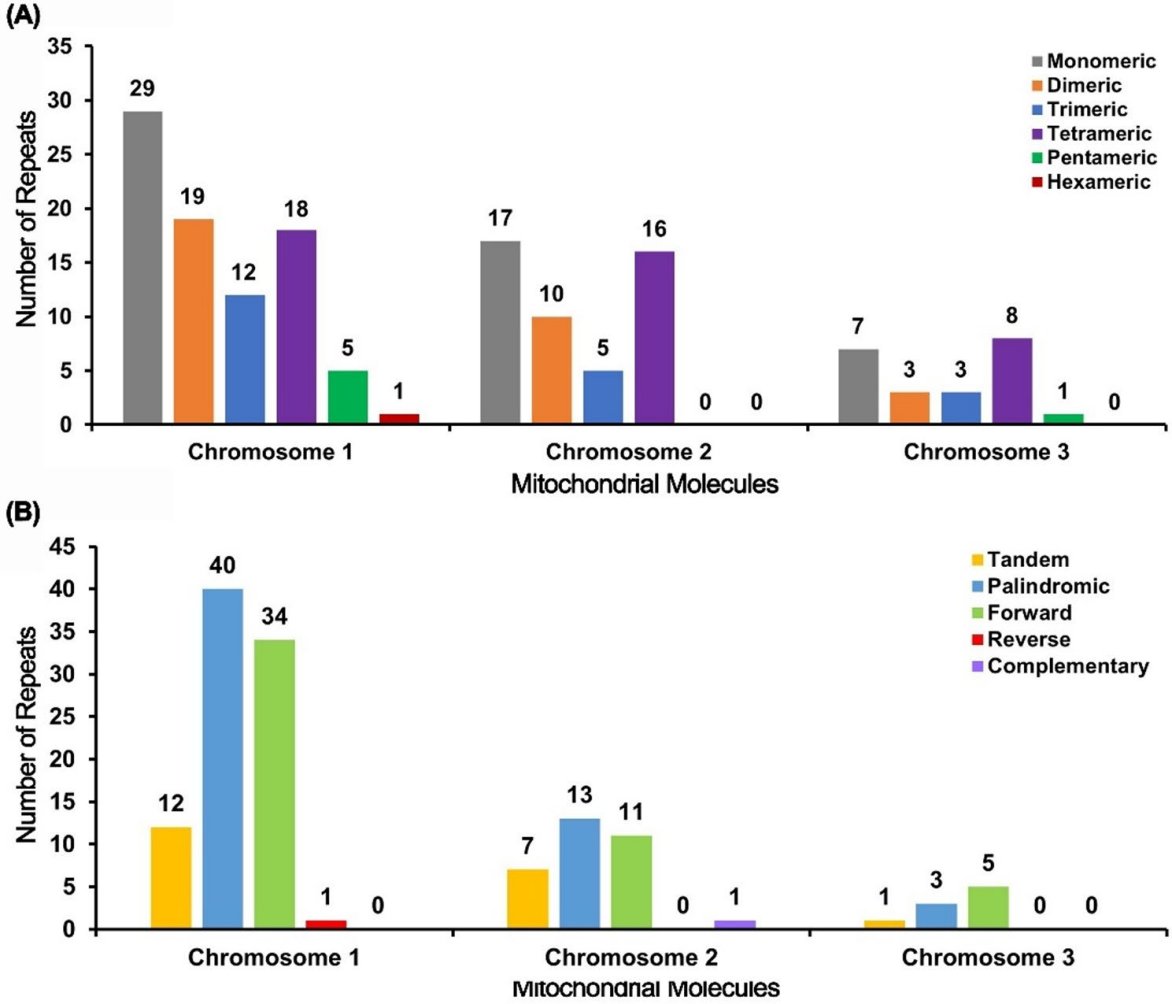
Repeat sequence analysis of the *M. tricuspidata* mitogenome. (A) The x-axis represents the type of SSRs while the y-axis represents the number of repeats. Each colored legend represents a different type of SSR: gray for monomer, yellow for dimer, blue for trimer, purple for tetramer, green for pentamer, and red for hexamer SSRs. (B) The x-axis displays the type of repeats, and the y-axis displays the number of repeats. The yellow, bule, green, red, and purple legends correspond to tandem, palindromic, forward, reverse and complementary repeats, respectively

Comprehensive repeat analysis of the tripartite *M. tricuspidata* mitogenome revealed distinct repetitive architectures across its three circular molecules. Chromosome 1 harbored the highest density of simple sequence repeats (SSRs), with 84 identified motifs predominantly composed of mono- (45.2%) and dinucleotides (27.4%). This chromosome contained 12 tandem repeats exhibiting > 75% sequence similarity, ranging from 15 to 24 bp in length. Dispersed repeat analysis detected 75 pairs ≥ 30 bp, dominated by palindromic (*n* = 40, 53.3%) and forward repeats (*n* = 34, 45.3%), with a single reverse repeat. Chromosome 2 possessed 48 SSRs showing comparable mono-/dinucleotide bias and featured 7 tandem repeats (12–39 bp) with > 88% similarity. Its 25 dispersed repeats ≥ 30 bp comprised palindromic (*n* = 13, 52.0%), forward (*n* = 11, 44.0%), and one complementary repeat. Chromosome 3 contained 22 SSRs and a single 19-bp tandem repeat (88% similarity), while dispersed repeats ≥ 30 bp totaled 8 pairs (palindromic: *n* = 3, 37.5%; forward: *n* = 5, 62.5%). Global analysis demonstrated non-random distribution: Chromosome 1 accounted for 54.5% of total SSRs and 69.4% of dispersed repeats ≥ 30 bp, underscoring its structural complexity. Size stratification of dispersed repeats revealed distinct molecular preferences: Forward repeats peaked at 30–49 bp (76.5% of total), while palindromic repeats dominated the 50–299 bp range (92.9%). Notably, Chromosome 2 contained the largest single dispersed repeat (276 bp, palindromic; E-value = 3.13e-157). Comparative analysis of repeat types across molecules showed consistent predominance of palindromic (56/108, 51.9%) and forward (50/108, 46.3%) configurations, with reverse and complementary repeats collectively representing < 2%. SSR composition analysis indicated universal A/T-richness, with mononucleotide (A)n/(T)n repeats constituting 48.7% of all SSRs. Tetranucleotide repeats (31.8%) were enriched in motifs containing AT/TA dimers (e.g., TTAA, AATA). The aggregate repeat landscape, visualized through multi-parameter histograms, demonstrates molecule-specific repetitive architectures that potentially influence mitogenome organization, where Chromosome 1 exhibits significantly higher repeat density (SSRs: 0.42/kb; dispersed repeats: 0.37/kb) compared to Chromosomes 2 (0.38/kb; 0.20/kb) and 3 (0.36/kb; 0.15/kb) (Fig. 4, Table S2, S3 and S4).

### DNA transfer from Chloroplast to mitochondrion

**Fig. 5:**
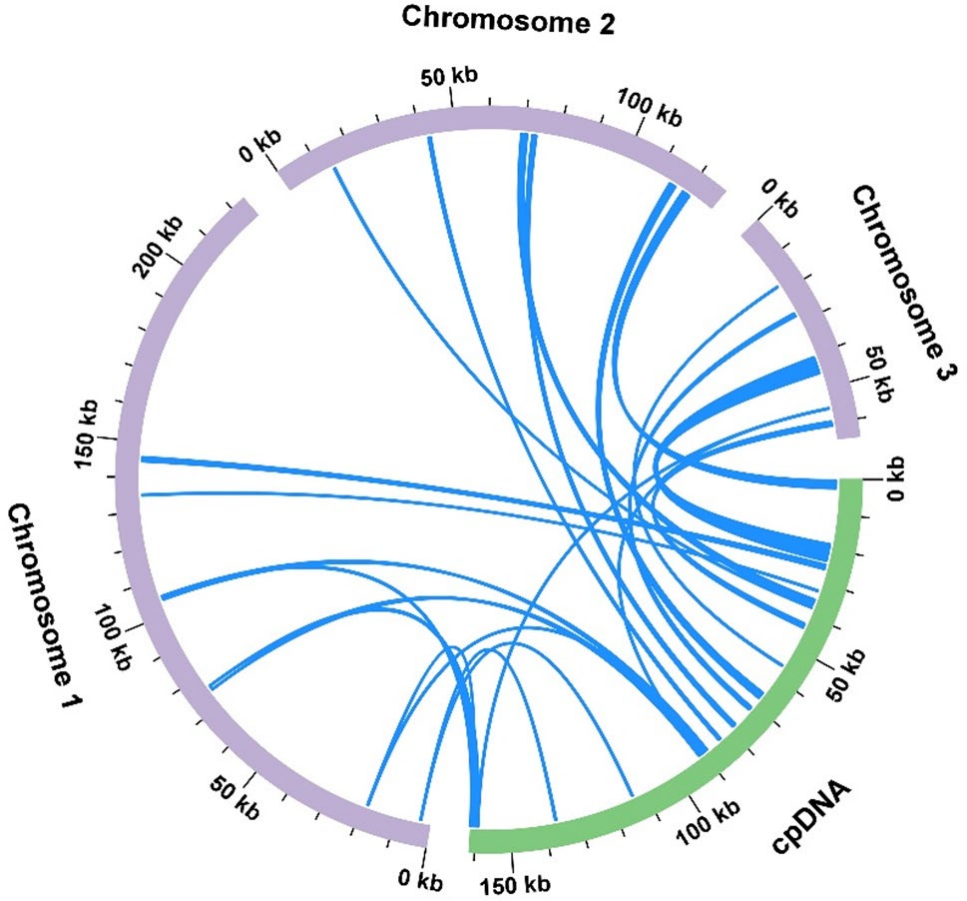
The gene transfers that occurred between the chloroplast and mitogenomes of *M. tricuspidata*. The purple and green arcs denote the mitochondrial and chloroplast genomes, respectively, while the blue lines connecting the arcs represent homologous genome segments that were transferred between the two organelles

The chloroplast genome was also sequenced, assembled, and annotated (Fig. S1). Analysis of chloroplast-to-mitochondrion DNA transfer identified 19 mitochondrial plastid DNA (MTPT) fragments with a combined length of 17,980 bp, representing 4.31% of the *M. tricuspidata* mitogenome (Fig. 5; Table S5). The longest fragment (MTPT15) spanned 4,728 bp. Annotation of these MTPTs revealed 11 intact chloroplast-derived genes, comprising five protein-coding genes (*petA*, *psbA*, *psbD*, *rpl23*, *rps19*) and six transfer RNA (tRNA) genes (*trnD-GUC*, *trnI-CAU*, *trnM-CAU*, *trnN-GUU*, *trnP-UGG*, *trnW-CCA*).

### RNA editing events in mitochondrion

**Fig. 6:**
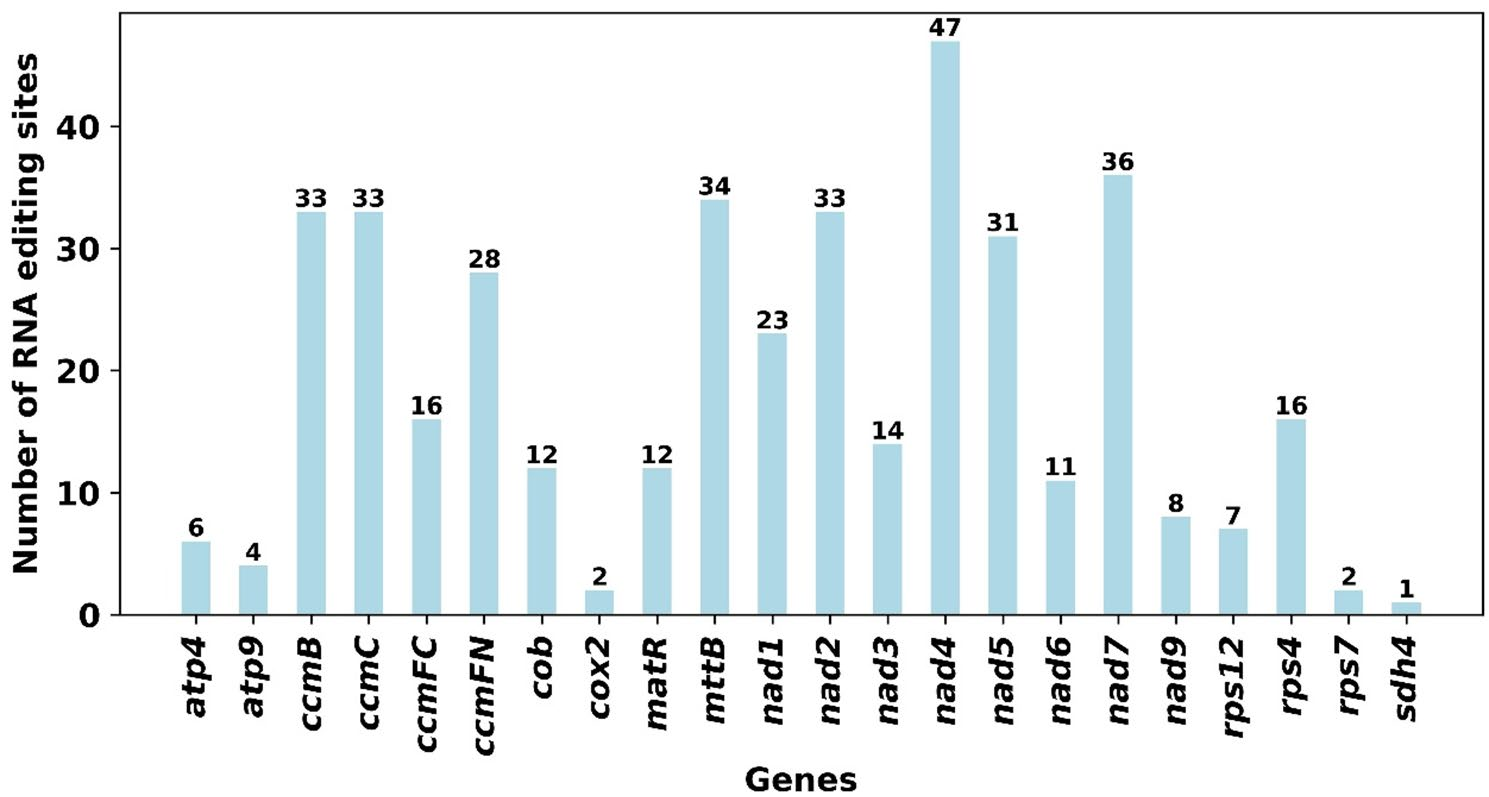
The Number of RNA editing sites predicted in PCGs of *M. tricuspidata* mitogenome

Prediction of RNA editing events using the Deepred-mt suite (cutoff = 0.9) identified 409 C-to-U editing sites among the 28 mitochondrial protein-coding genes (PCGs) (Fig. 6). Gene-specific analysis revealed significant variation in editing frequency. The *nad4* gene contained the highest number of sites (*n* = 47), followed by *nad7* (*n* = 36). Conversely, only one editing site was detected in *sdh4*. No RNA editing sites were predicted for the *atp1*, *atp6*, *atp8*, *cox1*, *cox3*, or *nad4L* genes. Critically, all 409 predicted editing events corresponded exclusively to C-to-U conversions (Table S6).

### Phylogenetic, positive selection and comparative synteny analysis

**Fig. 7:**
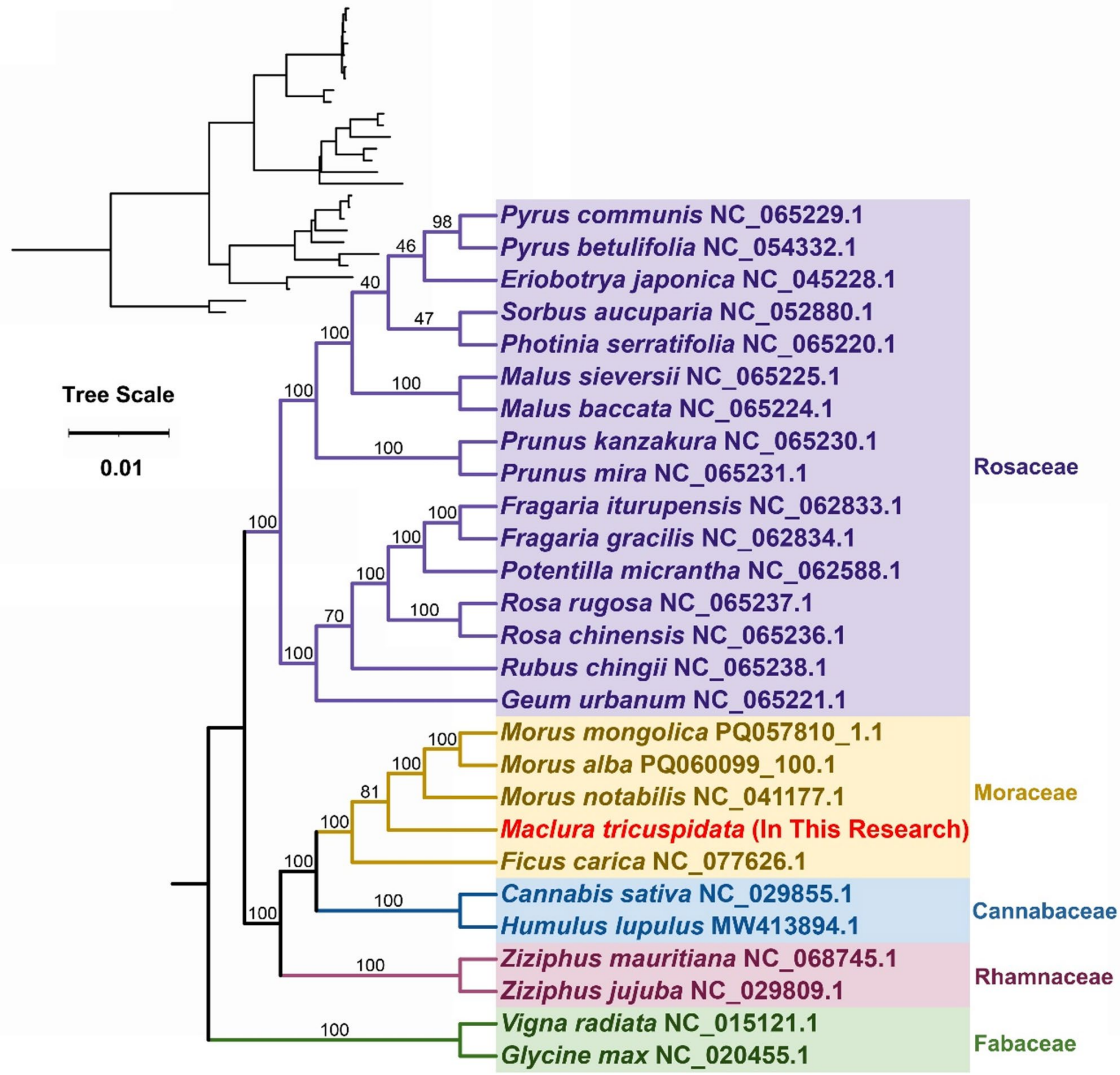
The phylogenetic relationships of *M. tricuspidata* with other closely related species. The Neighbor-Joining tree was constructed based on the sequences of 24 conserved PCGs. Colors indicate the families that the specific species belongs

Mitogenomes of 27 angiosperm species representing five families were retrieved from the NCBI database (Table S7). Phylogenetic reconstruction using 24 conserved mitochondrial PCGs (*atp*1, *atp*4, *atp*6, *atp*8, *atp*9, *ccm*B, *ccm*C, *ccm*FC, *ccm*FN, *cob*, *cox*1, *cox*3, *mat*R, *mtt*B, *nad*1, *nad*2, *nad*3, *nad*4, *nad*4L, *nad*5, *nad*6, *nad*7, *nad*9, *rps*4). The results revealed that *M. tricuspidata* belongs to the mulberry family Moraceae within the order Rosales and is closely related to *Ficus carica* and *Morus notabilis* (Fig. 7). This result aligns with the APG IV classification system, confirming the consistency of mitochondrial gene-based phylogeny.

**Fig. 8:**
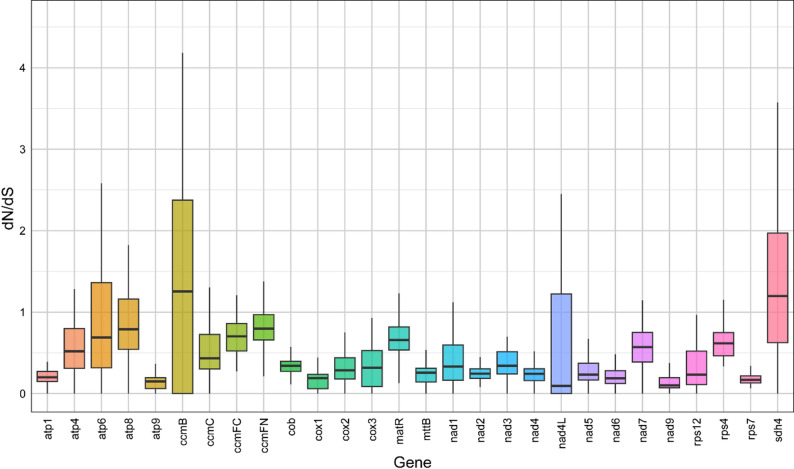
Distribution of dN/dS ratios across mitochondrial genes. Boxplots show the pairwise dN/dS values for each shared mitochondrial protein-coding gene among the 16 species. The box represents the interquartile range, the internal line indicates the median, and the whiskers extend to the minimum and maximum values

To assess selective pressures acting on protein-coding genes (PCGs) in *M. tricuspidata* and closely related species (Table S7), the ratios of nonsynonymous (dN) to synonymous (dS) substitutions (dN/dS) were calculated. We compared 28 mitochondrial PCGs from *M. tricuspidata* with their orthologs from 16 other species to estimate dN/dS values (Fig. 8). Gene-specific substitution analysis revealed a broad range of dN/dS ratios, from 0.03 in *atp8* to 7.87 in *sdh4*. The genes *ccmB* and *sdh4* displayed the highest mean dN/dS values (1.45 and 1.62, respectively), both exceeding 1, indicating the action of positive selection during evolution. In contrast, the majority of genes exhibited dN/dS values below 1 in most species, consistent with the prevalence of purifying selection. Notably, *nad9* and *atp9* showed the lowest average dN/dS values (0.158 and 0.159, respectively), which were consistently less than 1.0 across all species, reflecting strong functional constraints and high evolutionary conservation.

**Fig. 9:**
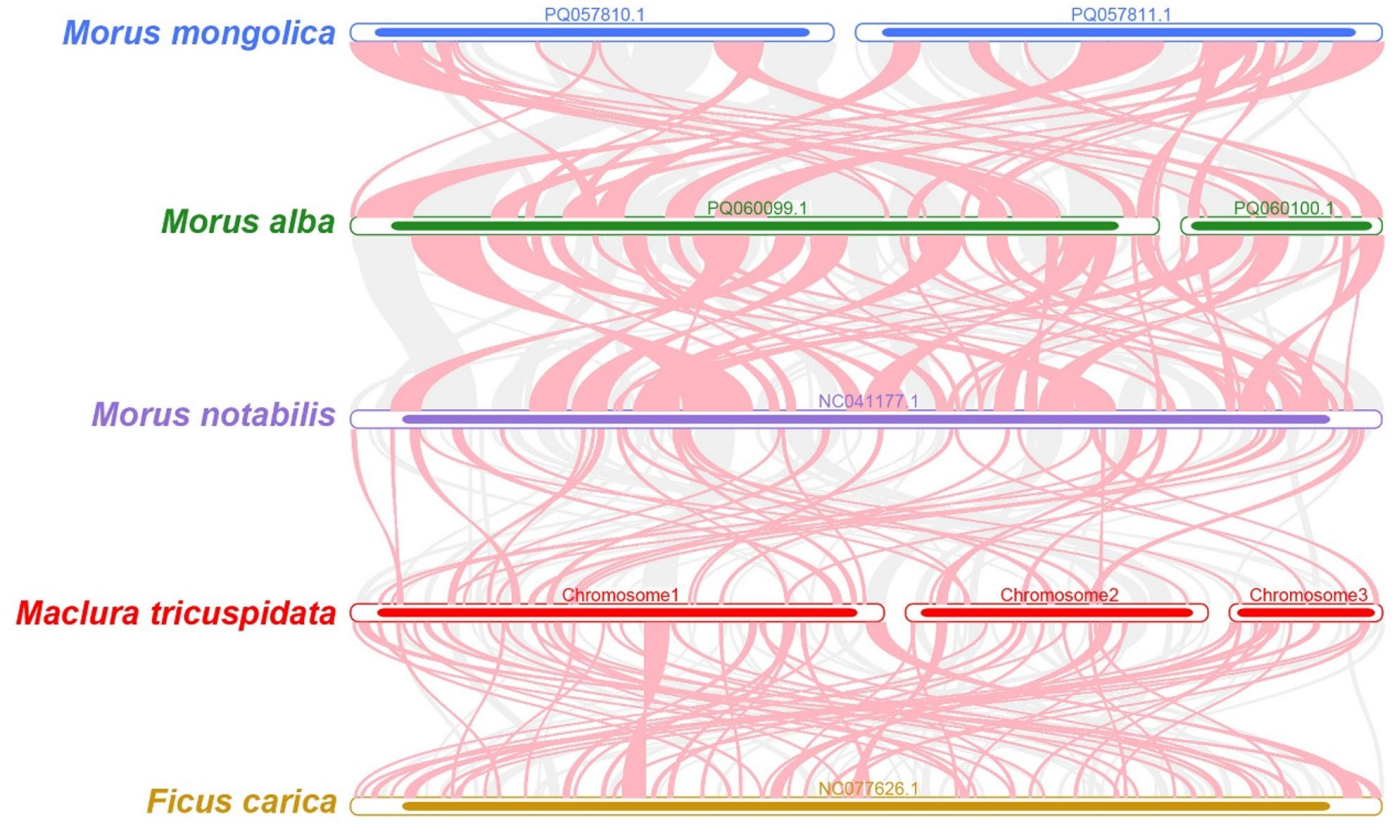
Mitogenome Multiple Synteny Plot of *M. tricuspidata* and closely related species. The bars on the graph indicate the mitogenomes of the species, while the ribbons depict the homologous sequences between adjacent species. The red areas highlight where inversions occurred, while the gray areas indicate regions with strong homology

Comparative synteny analysis of mitogenomes from *M. tricuspidata* and four related species (*Ficus carica*, *Morus mongolica*, *Morus alba*, *Morus notabilis*) identified numerous collinear blocks (Fig. 9). However, the length of these blocks was relatively small, with the largest block measuring 8,687 bp in length and exhibiting 98.285% identity between chromosome 2 of the *M. tricuspidata* and *Morus notabilis* mitogenomes. Substantial rearrangement of collinear blocks was observed across mitogenomes, indicating extensive genomic reorganization in *M. tricuspidata* relative to its close relatives and highlighting structural divergence. Additionally, specific regions of the *M. tricuspidata* mitogenome showed no homology to other species, suggesting these sequences are unique to this genome.

## Discussion

Mitochondria serve as indispensable energy factories in eukaryotic cells, generating ATP essential for cellular metabolism [29]. Plant mitogenomes exhibit exceptional structural plasticity compared to their animal counterparts, ranging from 190 kb to 11.3 Mb with complex configurations beyond canonical circular molecules [3, 10, 30]. Our assembly reveals a branched multi-chromosomal architecture in the *Maclura tricuspidata* mitogenome, distinct from the predominant single-circular conformation observed in many graminaceous species like *Oryza sativa* [31], *Elymus sibiricus* [32], and sugarcane cultivars [33]. Instead, it parallels the multibranched structure reported in *Populus deltoides* [6], comprising three circular chromosomes (Chromosome 1: 224,063 bp; Chromosome 2: 128,014 bp; Chromosome 3: 64,724 bp) totaling 416,801 bp. The overall GC content (44.94%) aligns closely with conserved values in higher plants, mirroring *Lolium perenne* (44.1%) [34] and *Hordeum vulgare* (44.2%) [35]. Annotation identified 28 protein-coding genes (24 core, 4 non-core including *rps4*, *rps7*, *rps12*, and *sdh4*), 18 tRNAs, and 3 rRNAs, suggesting functional conservation despite structural divergence.

Plant mitogenomes are rich in repeat sequences, our comprehensive analysis of the *M. tricuspidata* mitogenome revealed significant heterogeneity in repetitive element architecture across its three chromosomes, aligning with the recognized dynamism of repeats in plant mtDNA evolution [30, 36–39]. Chromosome 1 exhibited exceptionally high densities of both SSRs (0.42/kb) and dispersed repeats (0.37/kb), dominated by A/T-rich mono-/dinucleotides and palindromic/forward configurations, suggesting it as a primary hotspot for potential recombination-mediated structural rearrangements [30, 37, 39]. The predominance of palindromic (51.9%) and forward (46.3%) repeats across all chromosomes mirrors their established role in facilitating homologous recombination and isomerization in plant mitochondria [30, 38, 39], potentially explaining the observed structural complexity. The AT-rich tetranucleotide motifs (e.g., TTAA) and the large palindromic repeat (276 bp on Chr2) further emphasize the repeat expansion contributing to genomic size variation [40]. This non-random repeat distribution, particularly the complexity of Chromosome 1, likely underpins mitogenome plasticity and influences evolutionary trajectories [30, 41], while the characterized SSRs provide valuable markers for future phylogenetic and population studies in *Maclura* and its closely related genera within the Moraceae family [42, 43].

RNA-editing is a posttranscriptional process that occurs in the cp. and mt genomes of higher plants, contributing to the better folding of proteins [44]. In this study. We identified 409 C-to-U RNA editing sites across 28 mitochondrial PCGs in *M. tricuspidata* aligns with the established prevalence and functional significance of this post-transcriptional modification in plant organellar genomes [7, 44–46]. While the total number falls within the reported angiosperm range (typically 200–700), it exceeds counts in some species like *Carex breviculmis* (90) and *Suaeda* (216), suggesting species-specific variation consistent with observations in *Arabidopsis* (441), rice (491), and *Rehmannia chingii* (579) [31, 47]. The striking heterogeneity in editing density among genes—ranging from 47 sites in *nad4* to none in *atp1*, *atp6*, *atp8*, *cox1*, *cox3*, and *nad4L*—mirrors findings in other plants and underscores potential gene-specific regulatory requirements or evolutionary trajectories. The exclusive occurrence of C-to-U conversions, a universal feature in angiosperm mtDNA [46, 48], reinforces the conservation of the underlying biochemical machinery. The substantial editing burden on core respiratory complexes (e.g., *nad4*, *nad7*) likely optimizes protein function [7, 44–46]. Given the demonstrated link between RNA editing and stress adaptation [49], the characterized sites provide a foundation for exploring their potential adaptive significance in *M. tricuspidata*, while the absence of editing in specific genes warrants further investigation into their expression regulation and stability.

Unlike plastid and nuclear genomes, mitogenomes are rarely used in phylogenetic analyses of higher plants due to the low mutation rate, frequent genome rearrangement, and foreign DNA integration [50, 51]. Phylogenetic reconstruction based on 24 conserved mitochondrial PCGs robustly positions *M. tricuspidata* within the Moraceae family (Rosales order), demonstrating close evolutionary affinity to *Ficus carica* and *Morus notabilis*. This topology aligns precisely with the APG IV classification system, affirming the utility of mitochondrial PCGs for resolving angiosperm phylogeny at the family and genus level [52]. However, extensive collinearity analysis with four closely related Morus species and *Ficus carica* reveals striking structural divergence within the Moraceae mitogenomes. While numerous homologous collinear blocks were identified, their small size and drastically rearranged order, coupled with the presence of *M. tricuspidate* specific regions lacking homology, indicate exceptionally high rates of mitochondrial genomic rearrangement in this lineage compared to its relatives. This juxtaposition—conserved protein-coding sequences preserving deep phylogenetic signals versus highly dynamic genome architecture promoting lineage-specific structural evolution—highlights the complex, multi-layered nature of mitogenome evolution in angiosperms. The observed structural plasticity underscores the limitations of relying solely on gene sequences for understanding mitogenome evolution and emphasizes the need to integrate structural analyses, providing a framework for future investigations into mitogenome dynamics within Moraceae.

The dN/dS analysis and comparative genomic studies provide valuable insight into the evolutionary dynamics of plant mitochondrial genomes [53]. Consistent with previous reports [54, 55], the majority of protein-coding genes (PCGs) in the *M. tricuspidata* mitogenome evolved under purifying selection (dN/dS < 1), indicating evolutionary conservation of these genes. In contrast, *ccmB* and *sdh4* were found to be under positive selection, a notable exception that underscores their potential role in adaptive evolution. Genes exhibiting elevated dN/dS ratios, as observed here, are of particular interest for understanding species-specific adaptation and molecular evolution [56].

As the first mitogenome resource for *M. tricuspidata*—an economically significant species prized for bioactive flavonoid and xanthone biosynthesis—this comprehensive characterization establishes critical foundations for elucidating Moraceae evolutionary dynamics, deciphering molecular mechanisms underlying its medicinal properties, and enabling advanced studies of organelle-nuclear crosstalk regulating stress adaptation and specialized metabolism, thereby providing essential genomic infrastructure to unlock this non-model species’ full biotechnological potential.

## Conclusions

This study presents the first complete assembly and annotation of the mitogenome of *M. tricuspidata*, revealing a 416,801 bp multi-chromosomal structure. Annotation identified 28 protein-coding genes (24 conserved core genes, 4 accessory genes), 18 tRNA genes, and 3 rRNA genes. Comprehensive analyses characterized repeat sequences, predicted 409 C-to-U RNA editing sites, detected chloroplast-derived DNA transfers, and confirmed the species’ phylogenetic position within Moraceae consistent with APG IV classification. Collectively, this foundational mitogenome resource enables critical investigations into plant mitochondrial evolution, energy metabolism, and the molecular basis of this species’ valued medicinal properties.

## Materials and methods

### Plant materials and DNA sequencing

Fresh tender leaves of *M. tricuspidata* were collected from healthy individuals in Laiwu District, Jinan, China (36.502773°N, 117.7283°E; 732 m altitude). Note: This species is exempt from collection permits under *China’s Wild Plant Protection Regulations* and the *National Key Protected Wild Plants List* (2021). High-quality genomic DNA was extracted from the newly grown leaves using a modified CTAB protocol [57]. The DNA integrity was verified by 0.8% agarose gel electrophoresis, and purity was assessed using a Nanodrop spectrophotometer (A260/A280 ratio 1.8-2.0, concentration > 50 ng/µL). Following the manufacturer’s guidelines for PacBio Sequel II platform, we constructed a PCR-free SMRTbell library with 20 kb insert size. In total, 85.2 Gb PacBio HiFi data were generated, comprising 4,409,919 total reads. The average read length was 19.32 kb, with an N50 length of 19.28 kb.

### Mitogenome assembly and annotation

Mitogenome assembly was performed using PacBio HiFi long-read sequencing data. The raw HiFi reads were de novo assembled using Flye [58] with default parameters, generating an assembly graph in GFA format. For cross-validation, an independent de novo assembly was concurrently performed using HiFiasm (v0.19.5) [59]. All contigs in FASTA format derived from this assembly were used to construct a BLAST database with makeblastdb. To identify contigs harboring mitochondrial sequences, we performed BLASTn searches (parameters: -evalue 1e-5 -outfmt 6 -max_hsps 10 -word_size 7 -task blastn-short) using known *Arabidopsis thaliana* mitochondrial genes as query sequences. The assembly graph was visualized using Bandage (v0.8.1) [60]. Contigs exhibiting significant BLASTn hits to mitochondrial genes were identified and extracted from the assembly graph to generate a draft mitogenome.

The mitogenome was annotated using a combination of automated and manual approaches. Protein-coding genes (PCGs) were predicted using GeSeq (v2.03) [61] with the mitoenomes of *Arabidopsis thaliana* (NC_037304), *Ficus carica* (NC_035616), *Morus alba* (NC_056531) and *Liriodendron tulipifera* (NC_021152.1) as reference sequences. To enhance the identification of splice sites and trans-splicing genes, annotation was additionally performed using the IPMGA tool (http://www.1kmpg.cn/ipmga/). Transfer RNAs (tRNAs) were identified using tRNAscan-SE (v2.0.11) [62] with default parameters. Ribosomal RNAs (rRNAs) were annotated using BLASTN (v2.13.0) [63] searches against a database of known plant mitochondrial rRNAs. Finally, all gene annotations within the mitogenome were manually curated and corrected using Apollo (v1.11.8) [64] to ensure accuracy.

### Codon usage bias analysis

All protein-coding genes (PCGs) were extracted from the mitogenome using Phylosuite (v1.1.16) [65]. Multidimensional codon usage analysis was performed with CodonW (v1.4.4; http://codonw.sourceforge.net) to calculate relative synonymous codon usage (RSCU) values.

### Comprehensive repeat element identification

Repeat elements were systematically detected through three complementary approaches. Microsatellites were identified by MISA (v2.1) [66] with thresholds set as mono- (≥ 10 repeats), di- to hexa-nucleotides (≥ 5 repeats); tandem repeats were analyzed using TRF (v4.09) [67] with parameters: match score = 2, mismatch penalty = 5, minimum repeat length = 50 bp; dispersed repeats were detected via REPuter [68] (minimum length = 30 bp, similarity ≥ 90%, considering both direct and palindromic repeats). Repeat abundance was quantified with Excel (2021), and genome-wide distribution patterns were visualized as a heatmap using Circos (v0.69.9) [69].

### Sequence transfer analysis and RNA editing site prediction

Using the complete chloroplast genome of *M. tricuspidata* as reference, we performed BLASTn (v2.13.0) [63] alignments with stringent parameters (-evalue 1e-10 -word_size 11 -reward 2 -penalty − 3) to identify high-confidence homologous regions and then visualized as multi-track Circos (v0.69.9) [69] plots to delineate inter-organellar DNA transfer hotspots. For RNA editing analysis, all mitochondrial protein-coding genes (PCGs) were subjected to C-to-U editing site prediction using Deepred-mt [70], a convolutional neural network (CNN)-based tool demonstrating enhanced accuracy over conventional methods; all predictions with probability scores > 0.9 were retained for subsequent analysis.

### Phylogenetic analysis

Phylogenetic analysis was performed based on mitogenome data of closely related species selected according to the phylogenetic relationships of the target organism. Conserved protein-coding genes were extracted using PhyloSuite software (v1.1.16) [65]. And then multiple sequence alignment was conducted with MAFFT (v7.505) [71], followed by maximum likelihood (ML) tree construction using IQ-TREE (v1.6.12) [72] with the parameter settings “--alrt 1000 -B 1000” to perform 1000 ultrafast bootstrap replicates and SH-aLRT tests for branch support evaluation. The resulting phylogenetic tree was visualized and topologically optimized using the ITOL platform (v6) [73], which significantly enhanced the resolution of phylogenetic relationships.

### Synonymous and nonsynonymous substitution ratio analysis

The ratios of nonsynonymous to synonymous substitution rates (dN/dS) for mitochondrial protein-coding genes (PCGs) were analyzed to assess selective pressures in *M. trucuspidata*. Homologous sequences were identified by comparing the *M. trucuspidata* mitogenome against those of 16 other species (Table S7) using BLASTn [63]. The shared PCGs were aligned with MAFFT (v7.310, https://mafft.cbrc.jp/alignment/software/), and their dN/dS values were calculated with the CODEML program in the PAML package [74]. The resulting distributions of dN/dS ratios across genes were visualized as boxplots using the ggplot2 package in R.

### Synteny analysis

Initially, genome-wide conserved homologous sequences were identified using BLASTn (v2.12.0) [63] with stringent alignment parameters (E-value cutoff ≤ 1e-5, word_size = 9, gap opening penalty = 5, gap extension penalty = 2, match reward = 2, mismatch penalty=-3). High-confidence syntenic blocks were systematically filtered with minimum length ≥ 500 bp and directional consistency > 80%. MCscanX (v1.1) [75] was then employed to construct multiple synteny maps, enabling multidimensional display of cross-species syntenic relationships.

## Supplementary Information

Supplementary Material 1: Table S1. Relative synonymous codon usage (RSCU) values across amino acids in the M. tricuspidata mitogenome. Table S2. SSRs in the mitogenome of M. tricuspidata. Table S3. Tandem repeat sequences in the mitogenome of M. tricuspidata. Table S4. Dispersed repeat sequences in the mitogenome of M. tricuspidata. Table S5. The homologous DNA fragment in the M. tricuspidata mitogenome. Table S6. RNA editing sites predicted in PCGs of M. tricuspidata mitogenome. Table S7. Information of 26 downloaded genomes.

Supplementary Material 2: Fig. S1. The chloroplast gene map of M. tricuspidata.

## Data Availability

PacBio raw sequencing reads were deposited in the NCBI Sequence Read Archive (SRA) under accession number SRR34853580 (publicly available), while the annotated chloroplast and mitogenomes were deposited in GenBank under accession numbers PX023083 (publicly available) and PX023084 (publicly available), respectively.
