# Supplementary material for: Assembly and comparative analysis of the complete mitochondrial genome of the Maclura tricuspidata
Source: BMC Genomics. 2026 Jan 5;27:129. doi: 10.1186/s12864-025-12491-z (PMC12870742; doi:10.1186/s12864-025-12491-z)
Supplement: Supplementary file 2 — Supplementary Material 2: Fig. S1. The chloroplast gene map of M. tricuspidata. [file 12864_2025_12491_MOESM2_ESM.pdf]

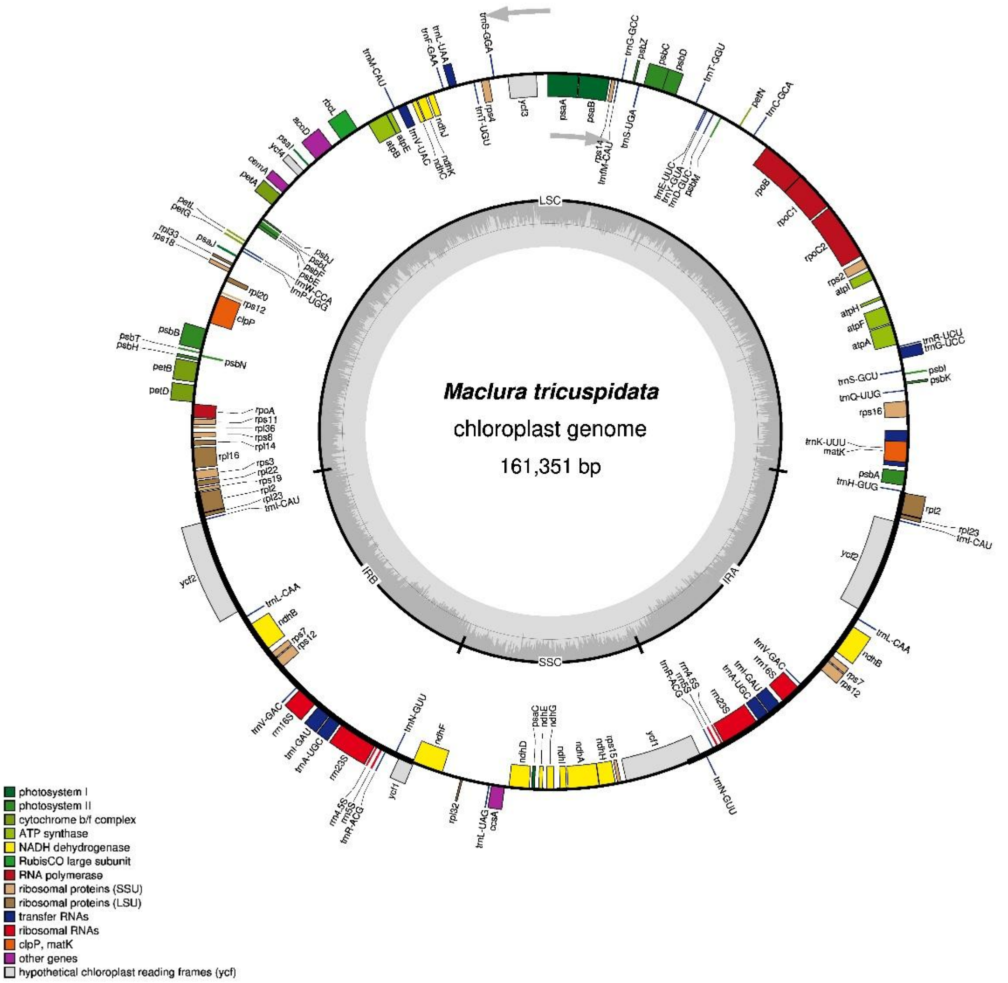


Fig. S1. The chloroplast gene map of *M. tricuspidata*. Genes located inside the circle are transcribed in a clockwise direction, while those outside the circle are transcribed counterclockwise.
